# The role of p21Waf1/CIP1 as a Cip/Kip type cell-cycle regulator in oral squamous cell carcinoma (Review).

**DOI:** 10.4317/medoral.18213

**Published:** 2013-02-05

**Authors:** Mario Pérez-Sayáns, José M. Suárez-Peñaranda, Pilar Gayoso-Diz, Francisco Barros-Angueira, José M. Gándara-Rey, Abel García-García

**Affiliations:** 1 PhD, DDS. Oral Medicine, Oral Surgery and Implantology Unit. Institute of Research of Santiago de Compostela (IDIS). Faculty of Medicine and Dentistry, Santiago de Compostela, Spain; 2 Anatomical Pathology Service, Clinical Hospital University of Santiago, Santiago de Compostela; 3 PhD, Department of Clinical Epidemiology, Clinical Hospital University of Santiago, Santiago de Compostela; 4 PhD; Molecular Medicine Unit- Galician Public Foundation for Genomic Medicine, Clinical Hospital University of Santiago, Santiago de Compostela; 5 MD, DDS, PhD; Oral Medicine, Oral Surgery and Implantology Unit, Santiago de Compostela, Spain; 6 MD, PhD. Oral Medicine, Oral Surgery and Implantology Unit. Institute of Research of Santiago de Compostela (IDIS). Faculty of Medicine and Dentistry, Santiago de Compostela, Spain

## Abstract

Oral Squamous Cell Carcinoma (OSCC) is biologically characterized by the accumulation of multiple genetic and molecular alterations that end up clinically characterized as a malignant neoplasm through a phenomenon known as multistep. The members of the Cip/Kip family, specifically p21Waf1/CIP1, are responsible for cell cycle control, blocking the transition from phase G1 to phase S. We made a search of articles of peer-reviewed Journals in PubMed/ Medline, crossing the keywords. The goal of this paper is to determine the relationship between p21Waf1/CIP1 expression and several clinical and pathological aspects of OSCC, their relationship with p53 and HPV, as well as genetic alterations in their expression pattern, their use as a prognosis market in the evolution of precancerous lesions and their roles in anticancer treatments. The results of p21WAF1/CIP1 expression in OSCC showed mixed results in terms of positivity/negativity throughout different studies. It seems that, although p21Waf1/CIP1 expression is controlled in a p53-dependent manner, coexpression of both in OSCC is not intrinsically related. Although the presence of HPV viral oncoproteins increases p21Waf1/CIP1 levels, the small number of studies, have forced us to disregard the hypothesis that HPV infected lesions that present better prognosis are due to a p21Waf1/CIP1–dependent control. The role of p21WAF1/CIP1 as cell-cycle regulator has been well described; however, its relationship to OSCC, the clinical and pathological variables of tumors, HPV and different treatments are not entirely clear. Thus, it would be very interesting to pursue further study of this protein, which may have a significant value for the diagnosis, prognosis and therapy of this type of tumors.

** Key words:**p21Waf1/CIP1, Cip/Kip type cell-cycle regulator, Oral squamous cell carcinoma (OSCC), p53, genetic alterations.

## Introduction

Oral Squamous Cell Carcinoma (OSCC) is biologically characterized by the accumulation of multiple genetic and molecular alterations that end up clinically characterized as a malignant neoplasm through a phenomenon known as multistep. The accumulation of damaged genetic material leads oral keratinocytes in an uncontrolled division of mutant cells (1).

**Figure 1:**
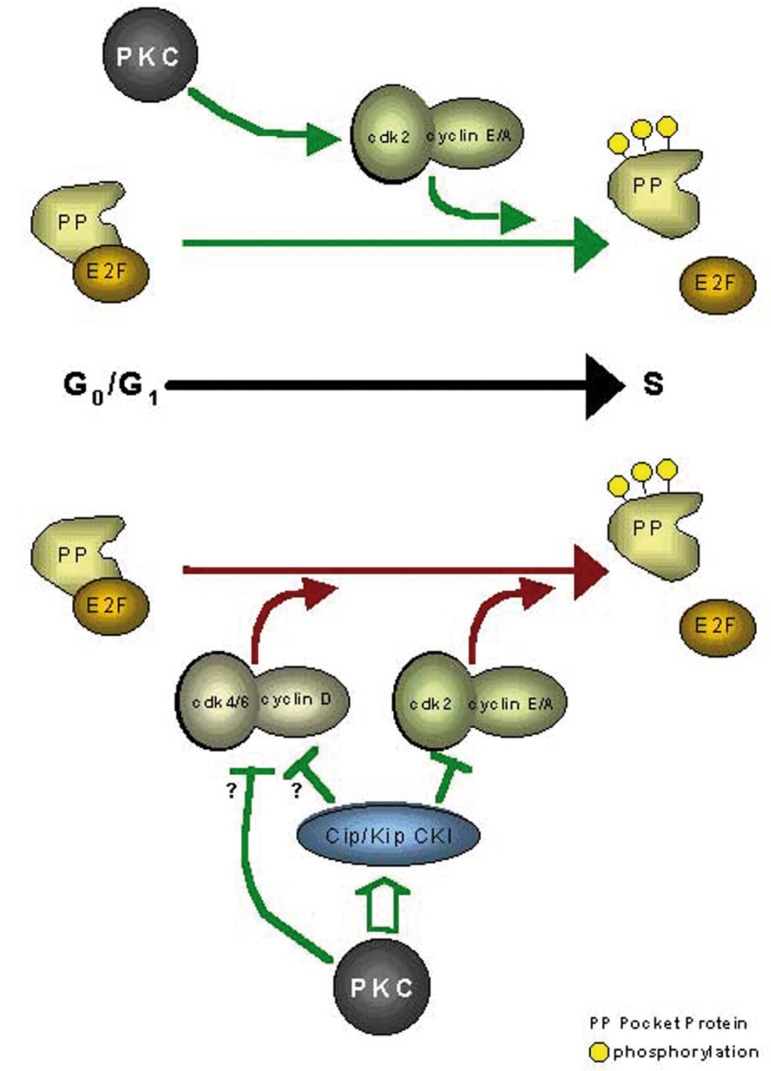
Model of the molecular mechanisms underlying PKC-mediated regulation of G1→S progression. The upper portion of the figure (above the black arrow indicating G0/G1→S progression) shows the consequences of PKC activation in early G1, while the lower portion (below the arrow) depicts events resulting from PKC activation in mid-to-late G1 phase (40).

Control of the cellular cycle is regulated by cyclins and cyclin-dependent kinases (CDK). The activity of these enzymes is restricted by the inhibiting action of two great (CDKI) inhibitor groups: the INK4 family, comprised by inhibitors p16, p15, p18 and p19; and the Cip/Kip family, comprised by p27, p57 and p21 (2). The members of the Cip/Kip family block activity of Cy-clin/CDK complexes, and specifically CDK2 in E/A-CDK2 cyclin complexes. These CDKIs block cell-cycle transition from G1 phase to S phase (3) (Fig. 1).

p21Waf1/CIP1 was initially identified as a 21 kDa protein that inhibited activation of cyclin/CDK (Cip1) complexe, later it was identified as an overexpressed gene in senescent cells, located at 6p21.1 (sdi1); furthermore p21 gene product, is transcriptionally activated by p53 in case of damage to DNA (WAF1). p21 is regulated by two different pathways, through a p53-dependent pathway (DNA damage leads to activation of p53 and upregulation of p21 causing cell cycle blockage in G1 phase with possible DNA repair or induction of apoptosis); and a p53-independent way, through which cell growth factors (platelet-derived growth factor, fibroblast and epidermal, but not the insulin growth factor that is able to induce p21 in p53-deficient cells in quiescence) (4).

We made a search of articles of peer-reviewed Journals in PubMed/ Medline, crossing the keywords. The goal of this paper is to determine the relationship between p21Waf1/CIP1 expression and several clinical and pathological aspects of OSCC, their relationship with p53 and HPV, as well as genetic alterations in their expression pattern, their use as a prognosis marker in the evolution of precancerous lesions and their roles in anticancer treatments.

## Model of the molecular mechanisms underlying PKC-mediated regulation of G1→S progression. The upper portion of the figure (above the black arrow indicating G0/G1→S progression) shows the consequences of PKC activation in early G1, while the lower portion (below the arrow) depicts events resulting from PKC activation in mid-to-late G1 phase (40).

The results of p21WAF1/CIP1 expression in OSCC showed mixed results in terms of positivity/negativity among different studies; although all agree that expression is entirely nuclear, unlike other CDKIs such as p16, in which nuclear/cytoplasm location results are variable (5-10). In  Table 1 and  Table 1 (continued), we can see the results of all the studies analyzing p21Waf1/CIP1 expression by immunohisto-chemistry (3,7,11-28) ( Table 1 and  Table 1 (continued)).

**Table 1:**
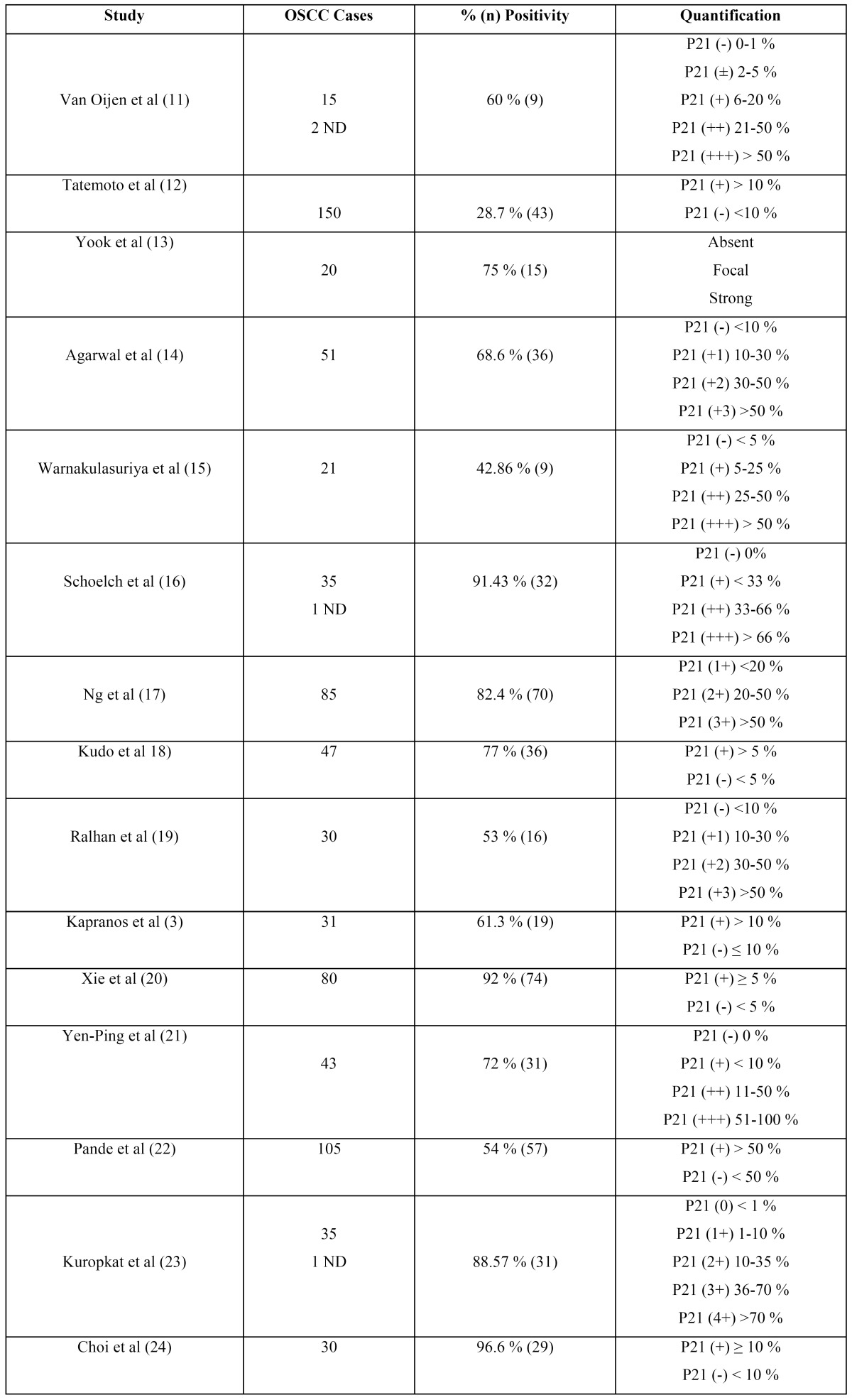
p21Waf1/CIP1 Immunohistochemical expression in OSCC.

**Table 1 (continued):**
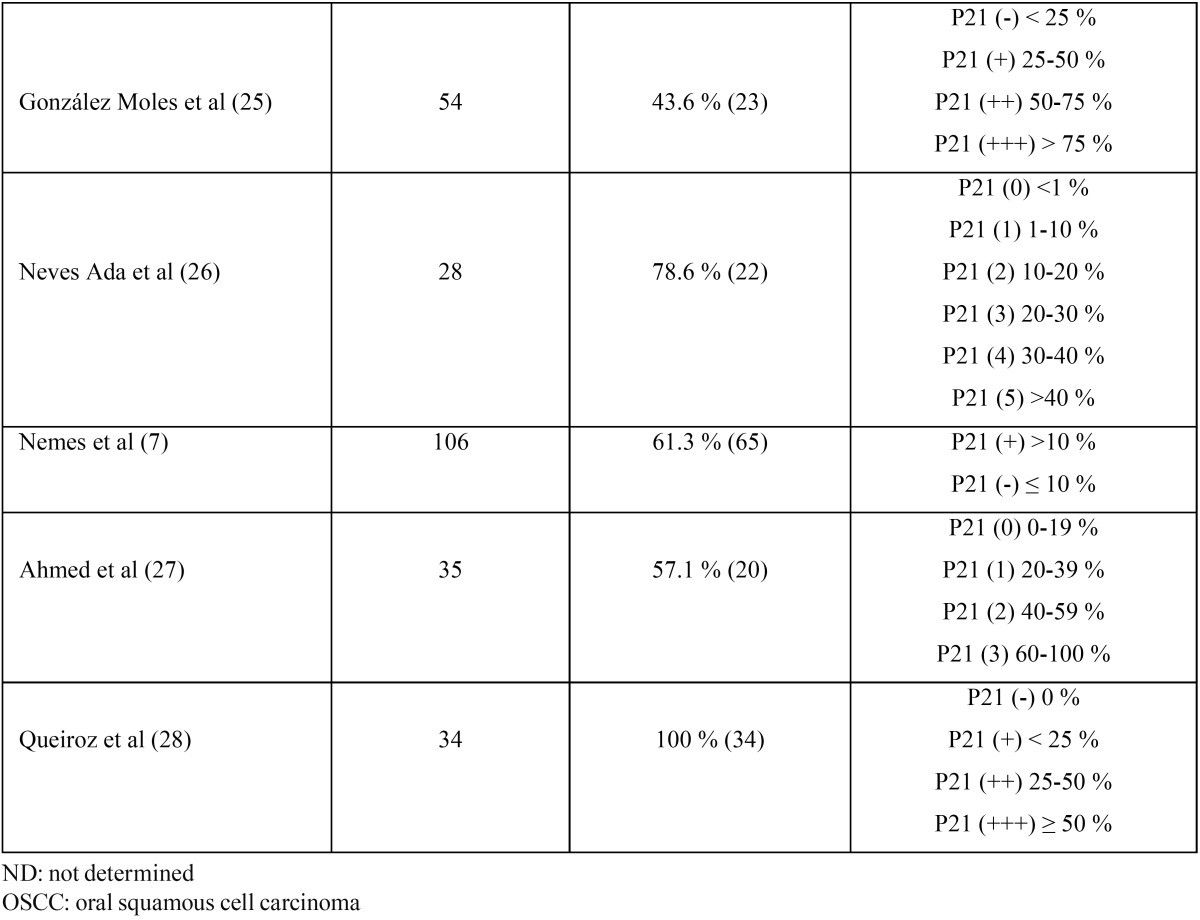
p21Waf1/CIP1 Immunohistochemical expression in OSCC.

As for p21 expression and its relationship with cell cycle control, Neves Ada et al. (26), found no correlation between the D1 and p21 cyclin expression, although its high expression in high-grade tumors supports its role in proliferative activity. However, forced stimulation of CDK4 causes sequestration of p21 in cell lines, increasing the risk of becoming OSCC, on the other hand the application of recombinant p21 in OSCC xenografts, has shown to cause delay in vivo tumor growth (29).

Regarding the relationship with clinical-pathological parameters, the results are highly variable, thus Fillies et al. (2) accounted for a relationship between loss of p21 expression and a decrease in overall survival, they also found an inverse correlation with tumor size. Tatemoto et al. (12), found no association between p21 expression with tumor stage, mode of invasion of tumor cells and the differentiation of the same. However, they found a correlation with the presence of metastases in lymph nodes, 25 cases (38,5%) of positive nodes and 18 cases (21.2%) of lymph nodes with negative tumor cells. According to Kapranos et al. (3), p21 shows positive expression in patients with HNSCC > 65 years, in chemotherapy-responsive tumors and stage III patients with a higher overall survival rate. According to Xie et al. (20), there is an inverse correlation between T classification and clinical stage, but not with N classification. Patients with p21 (+) tumors showed a greater disease-free interval than those patients with negative p21. The Hungarian group led by Nemes et al. (7), found a correlation between the expression of p21 and T3 and T4 stage tumors, positive lymph-node metastasis, cancer in advanced stages (III and IV) and tumors in the tongue and the retromolar trigone. On the other hand, Ng et al. (17), found no relation between p21 expression and TNM classification, but they reported a correlation in actively proliferating tumors, older patients and women. Yen-Ping et al. (21), reported no correlation between p21 expression and age, sex, oral habits, tumor location or TNM status, however they found a relationship between p21 (+) and a worse overall survival.

Other authors found no relationship between p21 expression and the clinical-pathological variables under study (22,25). Osaki et al. (30), found the same expression of p21 in well-controlled tumors than in lethal tumors, however they found no correlation with treatment failure or metastatic sites. According to González-Moles et al. (25), p21 expression is aberrant in all samples of non-tumoral adjacent epithelium and the absence of expression or decreased expression in tumors does not influence patient survival. Kuropkat et al. (23), found no relation between survival or time and recurrence. Yook et al. (13), found no correlation with any of the clinical-pathological parameters.

## p21Waf1/CIP1 and its relation with p53 expression

As mentioned above, p21 is regulated in two different ways; either through a p53-dependent pathway or a p53-independent one, so it is very important to establish the relationship between these two proteins in OSCC.

For van Oijen et al. (11) and Nemes et al. (7) the expression of p21 is independent of the presence of functional p53 in head and neck tumors (HNSCC) but its expression is related to the differentiation of tumors. In some tumors, p21 was expressed even in proliferating cells in which cyclin D1 was also expressed, suggesting that the inhibitory effect of p21 is offset by D1 cyclin expression in these tumors.

In this regard, Brennan et al. (4), found that the accumulation of nitric oxide synthase-2 (NOS2), which causes programmed cell death via p53 through p21 expression, from the 10.3 pmol NO min-1 mg protein-1, was sufficient to cause apoptosis by a p53-independent pathway, thanks to p21 expression. This data, together with the findings of Yook et al. (13), who reported that p21 expression was not associated with mutated p53 or with p53 protein overexpression, therefore supported the theory above, namely p21 expression by a p53-independent pathway.

Ng et al. (17) found no correlation between the p21 and p53 expression, on the other hand they found a relationship with mdm2 expression, an oncogene with self-regulating effect of the normal p53 expression. Similarly, Kudo et al. (18), found no association between p21 and p53.

If we address p53/p21 status and its relationship with clinical-pathological parameters, according to Kapranos et al. (3) patients with p53(-)/p21(+) tumors showed the highest survival rates, whereas p53(+)/p21(-) had a lower overall survival (3). Yen-Ping et al (21), found the highest 5-year survival rate in the p53 (+)/p21 (-) group and the lowest in the p53(+)/p21(+) group. According to Xie et al. (20) the disease-free period increased when the tumors were p53(-) as well as being p21(+). Yanamoto et al. (31), found that p53(+)/p21(+) tumors showed the worst clinical behavior in terms of survival. Agarwal et al. (14), in a total of 51 tumors, found that 58.8% were p53(+)/p21(+), while 17.7% of tumors were p53(-)/ p21(-), thus indicating the high heterogeneity of expression of these proteins in OSCC. Kudo et al. (18), described the highest percentage of survival for p5(-)/p21(+) tumors and the lowest for p53(+)/p21(+) tumors, as well as Yanamoto et al. (31).

## p21Waf1/CIP1 expression in precancerous lesions and the role of HPV 

In the dysplastic epithelium, p21 increases its expression as the degree of dysplasia increases. In OSCC, expression is variable, especially in poorly differentiated tumor areas. They found no association whatsoever with any clinical-pathological feature. Agarwal et al. (14), found a rising percentage of p21 expression as hyperplasia is transformed into dysplasia, associated with differentiation and proliferative activity. Kudo et al. (18), found among 24 epithelial dysplasias, 23 (96%) were positive for p21, compared to 77% (36 of 47) of OSCC. Furthermore, 79% of these dysplasias were p53(-)/p21(+) compared with 25% of OSCC. Queiroz et al. (28), found no statistically significant differences between p21 expression in normal oral epithelium, oral squamous papilloma and OSCC. Chang et al. (32), found a group of 53 verrucose leukoplakias, reporting p21 expression in 75% (40) of cases; 42% (22) developed OSCC in a period of three and a half years; 26% (14) were recurrent and 32% (17) were free of disease. Aberrant p21 positivity was associated with 80% progression to OSCC compared with 32% of recurrences. On the other hand, Hogmo et al. (33), found no relationship between p21 expression and risk assessment in terms of precancerous lesions.

As regards to the detection of HPV in OSCC and the expression of cell-cycle proteins, it seems that the E7 oncoprotein in high-risk HPV causes cell cycle dysregulation through the interaction of AP-1 complex transcription and CDKIs such as p27 and p21. Soares et al. (34), found that 11 out of 33 cases (33.33%) were positive for HPV18 (81.81%) or HPV16 (18.19%). Of these, p21 expression was positive in 5 of 11 cases(45.45%); they found no statistically significant association.

## Genetic alterations in p21Waf1/CIP1 expression pattern

As regards to the genetic alterations of p21 in OSCC, Ralhan et al. (19), described polymorphism at codon 149 (A→G) in 11 of 30 premalignant lesions (37%) (7 hyperplastic lesions and 4 dysplastic lesions) and in 11 out of 30 OSCCs (37%); being statistically significant compared to normal oral mucosa. It appears that this polymorphism is more common in precancerous lesions (10 of 11) and in OSCC (11 of 11) with weak p53 in lesions with mutated p53, suggesting that this polymorphism may affect the p53 pathway and play an important role in tumorigenesis. Gomes et al. (35), studied the relationship between p21WAF1/C98A polymorphism and its genotypes in OSCC. Thus, genotype CA showed a relative risk of malignant transformation 1.57 compared to CC genotype. Similarly, they showed that the heterozygous genotype has a higher immunopositivity CA (mean 56.75) and CC (mean 37.65) in a statistically significant way.

The frequency of mutations (transitions and transversions) in exon 2 of p21, according to Ibrahim et al. (36), ranges from 14-43% depending on the origin of tumors, being higher for Sudanese toombak-dippers tumors. In any case, the loss of 9p21 and its relationship with clinical-pathological parameters, can only be estimated in the context of the complex pattern of genomic imbalances that accompanies loss of chromosomes in the examined tumors.

## The role of p21Waf1/CIP1 in anticancer treatments

The induction of differentiation and anticancer agents remains today as the most appropriate strategy in cancer treatments, although in clinical practice results are insufficient. The mechanism of action of these molecules and agents in relation to p21 is not fully understood. According to Yoneda et al. (37), vesnarinone, a differentiation inducing agent inhibits cell growth and induces accumulation of cells in G1 phase without taking into account the existence of mutations in p53. Vesnarinone suppresses p21 promoter activity but produces p21-mRNA stabilization during a long period of time, therefore p21 protein expression is induced several hours after drug administration. The induction of p21 blocks cyclin E and suppresses the activity of the cyclin E/CDK2 complex, inhibiting pRb phosphorylation.

The mechanism of action of 5-FU (5-fluorouracil) and γ-rays, consists of an apoptotic effect of tumor cells yet in a p53 and p21 independent manner. According to Yoneda et al. (38), although p21-mRNA levels are increased with both agents, p21 protein was expressed only in parallel with increasing doses of radiation but not with 5-FU. According to this same group, in the case of TGF-β, there was an increase in p21 promoter activity in 7 of 9 cell lines in OSCC, while p21 protein expression increased sharply. However, when cells are transfected with Mn-SOD (Manganese Superoxide Dismutase), a potent proapoptotic agent derived from the action of various anti-tumoral agents such as 5-FU, PLM (a bleomycin derivative), CDDP (cis-diamminedichloroplatinum) and γ-rays, which after transfection of Mn-SOD, p21 expression increases dramatically inducing apoptosis of OSCC cell lines by p53-independent pathway.

These same results, in which there is an increase in p21 expression with cell-cycle blockage, have been described for perifosine (a new alkylphospholipid) , phenoxodial (a new isoflavone), EGCG (epigallocatechin-3-gallate, a polyphenol from green tea), SFN (sulforaphane) and also for cyclosporine A, growth inhibitory doses of EGF and X radiation (39).

As we have seen, the role of p21WAF1/CIP1 as cell-cycle regulator has been well described; however, its relationship to OSCC, clinical and pathological variables of the tumors, HPV and different treatment modalities are not entirely clear. Thus, it would be very interesting to pursue further study of this protein, which may have a significant value in terms of diagnosis, prognosis and therapy in this type of tumors.
